# Brain Oxygenation During Exercise in Different Types of Chronic Lung Disease: A Narrative Review

**DOI:** 10.3390/sports13010009

**Published:** 2025-01-08

**Authors:** Stella Kritikou, Andreas Zafeiridis, Georgia Pitsiou, Ioannis Gkalgkouranas, Leonidas Kastritseas, Afroditi Boutou, Konstantina Dipla

**Affiliations:** 1 Laboratory of Exercise Physiology and Biochemistry, Department of Sport Science at Serres, Aristotle University of Thessaloniki, 62122 Serres, Greece; kstyliana@phed-sr.auth.gr (S.K.); zafeirid@phed-sr.auth.gr (A.Z.); galgouranas@gmail.com (I.G.); lkastri@phed-sr.auth.gr (L.K.); 2 Department of Respiratory Failure, G. Papanikolaou Hospital, Aristotle University of Thessaloniki, 57010 Thessaloniki, Greece; gpitsiou@auth.gr; 3 Department of Respiratory Medicine, Ippokrateio Hospital of Thessaloniki, 54642 Thessaloniki, Greece; afboutou@yahoo.com

**Keywords:** brain oxygenation, exercise, lung disease, near-infrared spectroscopy, dyspnea, exercise intolerance, fatigue, Chronic Obstructive Pulmonary Disease, Interstitial Lung Disease, Idiopathic Pulmonary Fibrosis, Pulmonary Hypertension

## Abstract

Chronic lung diseases such as Chronic Obstructive Pulmonary Disease, Interstitial Lung Disease (ILD), and Pulmonary Hypertension (PH) are characterized by progressive symptoms such as dyspnea, fatigue, and muscle weakness, often leading to physical inactivity, and reduced quality of life. Many patients also experience significantly impaired exercise tolerance. While pulmonary, cardiovascular, respiratory, and peripheral muscle dysfunction contribute to exercise limitations, recent evidence suggests that hypoxia and impairments in cerebral oxygenation may also play a role in exercise intolerance. This narrative review (i) summarizes studies investigating cerebral oxygenation responses during exercise in patients with different types of chronic lung diseases and (ii) discusses possible mechanisms behind the blunted cerebral oxygenation during exercise reported in many of these conditions; however, the extent of cerebral desaturation and the intensity at which it occurs can vary. These differences depend on the specific pathophysiology of the lung disease and the presence of comorbidities. Notably, reduced cerebral oxygenation during exercise in fibrotic-ILD has been linked with the development of dyspnea and early exercise termination. Understanding the effects of chronic lung disease on cerebral oxygenation during exercise may improve our understanding of exercise intolerance mechanisms and help identify therapeutic strategies to enhance brain health and exercise capacity in these patients.

## 1. Introduction

Chronic lung diseases are becoming a serious global health crisis, as they contribute to leading causes of death and disability and are associated with decreased quality of life [1]. In 2017, over 544 million people, particularly older adults, suffered from chronic lung diseases [2], representing a significant increase of approximately 40% since 1990 [3]. Lung diseases can affect several components of the respiratory system, such as the airways, lung parenchyma, and pulmonary vasculature, leading to impaired gas exchange [4]. The most common types are chronic obstructive pulmonary disease (COPD), interstitial lung diseases (ILD), pulmonary hypertension (PH), asthma, lung cancer, and cystic fibrosis [5]. This review will focus on the first three types of disease.

COPD is characterized by progressive airflow limitations and chronic symptoms such as dyspnea, cough, and expectoration. Persistent abnormalities in the airways and/or alveoli have been associated with chronic inflammation, oxidative stress and disturbance between balanced enzymes (such as proteases and antiproteases). Pathophysiological changes in the lungs include increased dead space, ventilation-perfusion mismatch, impaired gas exchange, and hyperinflation that significantly worsen during exercise [6,7,8,9,10].

The ILDs are a heterogenous group of over 200 respiratory conditions that primarily affect the lung parenchyma, specifically the interstitium [11,12,13]. Progressive scarring and inflammation of lung tissue lead to impaired lung function and reduced gas exchange capacity. Idiopathic pulmonary fibrosis (IPF), one of the most common types of ILD, is characterized by chronic and progressive fibrosis of the alveolar spaces, resulting in profound limitations in oxygen transport and respiratory efficiency. Patients with ILD commonly present symptoms such as cough, dyspnea, and fatigue [14,15]. They exhibit an abnormal physiological pattern with varying degrees of inflammation and/or irreversible fibrosis between the alveoli and capillaries, which decreases the flexibility of the lungs and leads to a restrictive respiratory pattern [11,16,17]. These changes lead to a further decline in cardiorespiratory function, an early sense of dyspnea, impaired gas exchange, and low exercise performance [13,16,17,18,19].

The third type of disease, PH (defined as mean pulmonary artery pressure >20 mmHg measured by right heart catheterization), is a progressive and serious lung condition characterized by increased pressure in the pulmonary arteries, causing an inability of the heart to pump blood through the lungs, eventually leading to right heart failure [20]. There are five main PH types based on the underlying cause of abnormal pulmonary artery pressure: pulmonary arterial hypertension (PAH), PH due to left heart disease, PH due to lung disease and/or hypoxia, pulmonary artery obstructions (mainly Chronic Thromboembolic Pulmonary Hypertension, CTEPH), and PH with unclear or multifactorial mechanisms. Patients with PH also experience significant limitations to exercise due to dyspnea and deconditioning [20]. Despite the different pathologies, all these lung conditions are progressively characterized by deterioration in exercise tolerance.

Exercise intolerance is defined as a reduced ability to perform physical activities or exercise that would typically be expected for a person’s age and size. The symptoms associated with exercise intolerance, such as significant dyspnea and/or fatigue, are strong determinants of health-related quality of life in patients with lung disease. The pathophysiological mechanisms of exercise intolerance in chronic lung diseases are multifactorial, and primarily include impairments in pulmonary and cardiac reserve, as well as reduced function of both respiratory and peripheral skeletal muscles [21]. Recent studies have indicated that additional factors, such as the cerebrovascular system and limitations in dynamic cerebral autoregulation and cerebral oxygenation, can significantly contribute to the development of dyspnea and poor exercise tolerance [22,23,24]. Cerebral oxygenation plays a pivotal role in maintaining neuronal and glial homeostasis. Healthy brain functioning depends on matching the metabolic demands to the delivery of oxygen and nutrients [25]. During exercise, cerebral activity increases, and thus, cerebral perfusion and oxygenation should rise in brain areas involved with the specific task [26]. However, research suggests that the brain may reach a limited capacity to increase its oxygenation during exertion, which can hinder motor unit recruitment and muscle activation, signaling fatigue, and leading to the discontinuation of exercise [27,28].

Previous studies have examined the role of alterations in brain oxygenation and/or acute hypoxia in fatiguability, both in healthy individuals and in patients with various chronic diseases (coronary artery disease and gestational diabetes mellitus) [29,30]. However, there is limited information on how different types of chronic lung diseases affect brain oxygenation during exercise. The aim of this narrative review was to summarize the research findings on cerebral oxygenation during exercise in patients with chronic lung diseases, specifically COPD, ILD, and PH, and to compare the responses between healthy individuals and patients with these different disease types. Investigating the effects of chronic hypoxia on cerebral oxygenation during exercise in these patients may improve our understanding of the mechanisms underlying exercise intolerance and also help identify therapeutic strategies to enhance brain health in individuals with chronic lung diseases.

## 2. Methods

A review of the existing literature was conducted using the PubMed and Scopus databases to identify studies that assessed brain oxygenation during exercise in patients with COPD, ILD, and PH. The search strategy involved a combination of terms: “brain”, “cerebral”, “lung disease”, “chronic lung disease”, “Chronic Obstructive Pulmonary Disease”, “COPD”, “Interstitial Lung Disease”, “ILD”, “Idiopathic Pulmonary Fibrosis”, “Pulmonary Hypertension”, and “PH”, along with either “exercise” or “exercise-testing”, and the terms “brain oxygenation”, “Near-infrared spectroscopy”, and “NIRS”. The article search was performed independently by three authors between February and September 2024. Titles and abstracts were screened for relevance, and full-text articles, as well as manuscripts that cited these articles, were retrieved to determine eligibility for inclusion in the review. The inclusion criteria were as follows: (i) original research articles in English, (ii) studies involving human participants, (iii) patients with chronic lung diseases of the specified types, with and without co-morbidities, and (iv) assessment of cerebral oxygenation during exercise using NIRS.

## 3. Cerebral Oxygenation During Exercise: Mechanisms and Implications for Fatigue

Despite its small size, the brain demands approximately 20% of the oxygen consumed by the entire human body and about 15% of the cardiac output under normal resting conditions [31]. To preserve normal brain function and tissue integrity, it is essential to maintain the continuous delivery of oxygen and nutrients and the effective clearance of carbon dioxide (CO_2_) and cellular waste [32]. During states of increased cerebral activity, such as exercise, the cerebral metabolic demands significantly increase. To ensure oxygen availability, cerebral blood flow increases through the following synergistic adaptive mechanisms: (a) autoregulation (the ability of the cerebrovasculature to adapt to changes in perfusion pressure), (b) cerebral vascular reactivity to vasoactive stimuli (such as vascular dilation in response to CO_2_), (c) endothelium-dependent regulation/vasorelaxation, and (d) neurovascular coupling (e.g., the adaptations of cerebral blood flow in response to alterations in neural activity) [33]. Therefore, cerebral oxygenation reserve (i.e., the brain’s ability to maintain or increase oxygen supply to its tissues under conditions of increased demand) depends on the ability of the cerebral vasculature to augment blood perfusion in response to elevated metabolic demands associated with heightened neuronal activity in specific brain areas [34].

During exercise, cerebral activity increases, and oxygen and substrate demands are higher. Thus, the ability to provide sufficient oxygen delivery during exercise is crucial. As previously mentioned, sufficient oxygen delivery to the brain during exercise relies on two main factors: cerebral blood flow and the amount of oxygen in the blood [35]. Studies in healthy individuals have shown an increase in cerebral oxygenation in the prefrontal cortex (PFC) and motor-related areas during aerobic exercise [26]. In fact, cerebral blood flow and oxygenation increase even in the anticipation of exercise [36], possibly due to the activation of neurons involved in planning of the exercise [37,38]. On the other hand, it has been reported that reductions in cerebral oxygenation in areas such as the PFC during exercise could not only negatively affect cognitive function (e.g., impair executive function reaction time, thinking, and memory) [39,40,41] in both healthy individuals and those with chronic diseases, but also hinder the central nervous system (CNS) ability to fully activate the muscles required for continuation of exercise [28,29]. In healthy individuals, Rasmussen et al. (2010) reported that exhaustive exercise has similar effects on cerebral oxygenation alterations and fatigue markers, as exercise performed during acute hypoxia [42]. This finding suggests that reduced cerebral oxygenation may be a key factor contributing to central fatigue, thereby limiting exercise capacity [42].

### 3.1. Near-Infrared Spectroscopy (NIRS): A Non-Invasive Tool for Measuring Cerebral Oxygenation During Exercise

For assessing cerebral oxygenation and blood flow, several methods have been used. These methods have evolved over time from invasive (using intravenous catheters) to noninvasive (ultrasound, MRI, near-infrared spectroscopy—NIRS). Recent neuroimaging technologies (e.g., NIRS) made it possible to assess not only during resting conditions but also during dynamic conditions, such as during exercise or mental tasks. In this review, studies that used the NIRS technology have been examined. NIRS can detect quick fluctuations in cerebral oxygenation, and it is relatively unaffected by motion [43]. For this reason, it has been used during exercise. Briefly, the NIRS technology assesses relative changes in oxygenated (O_2_Hb), deoxygenated (HHb), and total (tHb) hemoglobin concentrations (μM·s^−1^), and provides information regarding tissue oxygen saturation (TSI, %) and cerebral activation in specific brain regions [44]. Changes in O_2_Hb (an index of O_2_ delivery in the local tissue) and hemoglobin difference (Hbdiff) serve as markers of tissue oxygenation, while HHb reflects the rate of tissue oxygen extraction. Lastly, tHb indicates changes in regional blood volume [45]. The NIRS parameters reflect the dynamic balance between oxygen demand and supply in the cerebral microcirculation. An increase in O_2_Hb and no change or a decrease in HHb concentration has been suggested to denote cortical activation [46]. In contrast, decreased O_2_Hb and increased HHb denote impaired oxygenation of the brain [46].

### 3.2. Factors Affecting Cerebral Oxygenation Response During Exercise: Intensity, Fitness, and Age

During exercise, the acute responses in brain oxygenation can vary depending on the type and intensity of exercise. Specifically, from rest to moderate intensity (25–75% $\dot{V}$O_2_peak) aerobic exercise, cerebral oxygenation typically increases [47]; however, at higher intensities near maximal exercise, hyperventilation can promote cerebral vasoconstriction and reduce cerebral blood flow [48,49,50]. For example, Bhambhani et al. (2007) [51] reported that during incremental exercise, cerebral oxygenation began to decline at the respiratory compensation threshold, possibly due to the reduction in cerebral blood flow and subsequent neuronal impairment. Further elaborating on this topic, Tsubaki et al. [26] showed variations in cerebral oxygenation across different exercise intensities (30%, 50%, and 70% $\dot{V}$O_2_peak).

Possible contributing factors that could also affect cerebral oxygenation response during exercise include fitness levels and age [52,53,54,55,56]. Briefly, aerobically trained individuals have been reported to exhibit a greater cortical O_2_Hb and tHb response than untrained individuals [57]. Nevertheless, even in trained individuals, exercise intensity above a certain threshold could lead to a point where the cerebral oxygen demand exceeds its supply, resulting in a reduction in brain oxygenation. However, aerobic training offers some protection by allowing for a higher exercise intensity before this critical point is reached, as the appearance of the ventilatory threshold occurs at a higher percentage of $\dot{V}$O_2_max [52]. Aging has also been associated with lower cerebral blood flow perfusion and oxygenation at rest and during exercise [54,55]. Puthon et al. [56] reported that even healthy older individuals exhibited a smaller rise in O_2_Hb and tHb compared to younger individuals during exercise. Interestingly, under hypoxic conditions, young individuals showed a larger reduction in prefrontal cortex O_2_Hb and an increase in HHb during exercise compared with older individuals. In contrast, prefrontal tHb showed similar relative changes from normoxia to hypoxia in both groups. The larger hypoxia-induced changes in O_2_Hb and HHb in young subjects might, therefore, reflect a greater imbalance between prefrontal cortex oxygen delivery and consumption in young people compared with older individuals during exercise in acute hypoxia [56]. Furthermore, Braz et al. (2016) reported that aging negatively impacts cerebral oxygenation and perfusion responses even during exercise near moderate intensities [58].

In summary, intensity (moderate: 30% to 70% $\dot{V}$O_2_peak or high >70% $\dot{V}$O_2_peak), fitness level (trained vs. untrained), and age (young vs. old) can influence the cerebral oxygenation responses during exercise [47,49,50,57]. When the brain does not receive enough oxygen, it might become less activated [22,59,60], resulting in a reduced ability to fully activate the muscles required for continuing the exercise. This may trigger the feeling of exhaustion, known as central fatigue, which could limit exercise capacity [42,61].

## 4. Cerebral Oxygenation During Exercise, Assessed Using NIRS, in Chronic Lung Diseases

### 4.1. Cerebral Oxygenation During Exercise in Patients with CHRONIC Obstructive Pulmonary Disease (COPD)

To our knowledge, only eight studies examined cerebral oxygenation during exercise in patients with COPD (*n* = 183 patients in total) (Table 1). Higashimoto et al. investigated the activation of different cerebral regions during exercise in COPD patients compared to controls. The O_2_Hb changes in the PFC during exercise were correlated with dyspnea (BORG dyspnea scale) in COPD and controls (r = 0.75, r = 0.78, and, respectively, *p* < 0.05) [62]. Oliveira et al. [63] and Higashimoto et al. [45] examined COPD patients with and without exertional desaturation. In the first study, the participants underwent two exercise protocols, one in a normoxic and one in a hyperoxic environment [63]. Vogiatzis et al. compared cerebral oxygenation and blood flow of COPD patients when exercising in room air, vs. under oxygen (100%) or normoxic heliox supplementation [64]. Furthermore, Andrianopoulos et al. compared cerebral oxygenation in COPD patients with and without cognitive impairment (CI) that exhibited mild to moderate exertional desaturation (SpO_2_ decline by 3–5%) [65]. Moreover, three studies examined cerebral oxygenation responses in COPD patients and COPD with comorbidities, such as heart failure [66,67,68].

Incremental exercise to exhaustion was performed in three studies [63,66,67], while steady state workload (at 75 of peak capacity) cycling was used in the other studies (*n* = 5) [45,62,64,65,68]. Cerebral prefrontal oxygenation was assessed in most studies, except in Higashimoto et al.’s studies, which monitored several cerebral areas (Table 1). Of the studies that examined cerebral oxygenation, five [45,62,63,66,67] reported an increase in brain oxygenation (O_2_Hb) during exercise, while one [65] observed that in COPD patients that were not oxygen-dependent at rest/exercise, cerebral oxygenation (TSI) remained unchanged from baseline throughout the exercise session. Furthermore, in the latter study, COPD patients with mild cognitive impairment preserved the capacity to oxygenate their brain during exercise to the same extent as patients without cognitive impairment. In the study, investigating the effects of air, normoxic heliox, and oxygen supplementation on cerebral oxygenation during exercise, the authors reported that cerebral oxygenation remained unchanged compared to rest in both air and heliox supplementation, and increased only during oxygen supplementation [64].

The degree of exercise desaturation seems to influence brain oxygenation responses. In further detail, Oliveira et al. [63] reported that COPD patients that present only exercise desaturation (not hypoxemic at rest, not on long-term oxygen therapy) exhibit lower cerebral oxygenation compared with non-desaturators. In that study, cerebral oxygenation in desaturators remained unaltered throughout exercise, whereas in non-desaturators, it increased progressively from rest to moderate intensity exercise. Oxygen supplementation improved brain oxygenation in desaturators, despite the unaltered cardiac output and mean arterial pressure. These findings suggested that an enhancement in CaO_2_ was possibly the main mechanism to these improvements, rather than the enhancement in central hemodynamics [63]. In addition, Higashimoto et al. reported that although prefrontal O_2_Hb increased in the non-hypoxemic COPD patients and in the control group during steady state exercise (at 40% of peak work), in hypoxemic COPD patients, O_2_Hb decreased during the initial phase of exercise (1–5 min) and then increased during the later exercise phase (8–10 min) [45]. Overall, pre-frontal O_2_Hb in the hypoxemic COPD patients were significantly lower than in the control and the non-hypoxemic COPD patients. Moreover, in hypoxemic patients, a rise in HHb (assumed to reflect impaired cerebral oxygenation) was observed during exercise, and this response in the dorsolateral area was correlated with dyspnea (r = 0.75, *p* < 0.05). In contrast, in control and non-hypoxemic patients, HHb (in the pre-motor cortex) decreased during exercise, signifying cortical activation. The responses in tHb were not significantly different among groups throughout the exercise session. In that study, oxygen supplementation also improved brain oxygenation in the hypoxemic group. In COPD patients, comorbidities, such as heart failure, also influenced brain oxygenation responses during exercise [66,67,68]. Specifically, in COPD patients with reduced ejection fraction, O_2_Hb remained stable or decreased in most of the patients (14 out of 18 patients) [66], whereas it increased in patients without HF. In another study by Oliveira et al., cerebral O_2_Hb levels in patients with COPD and HF remained at or below resting values from the beginning of exercise, despite preserved arterial saturation/oxygenation, whereas in patients with COPD without HF, O_2_Hb increased after 60% of peak power [67]. These abnormalities in patients with COPD-HF were associated with lower systemic mean arterial pressure and lower cardiac output, together with lower PaCO_2_. Thus, the negative impact of COPD (due to static or dynamic hyperinflation, V/Q mismatch), along with heart dysfunction (reduced preload and increased afterload) and vascular dysfunction, contribute to reduced brain oxygenation during exercise [68].

Three studies that examined cerebral deoxygenation responses (HHb) in COPD patients reported an increase during exercise [62,65,68]. It is not clear whether arterial desaturation contributes to the rise in HHb. Higashimoto et al. observed a decline in HHb in COPD patients without desaturation and in healthy individuals [45], whereas Andrianopoulos et al. did not observe a significant correlation between cerebral oxygenation and pulse oximetry levels (SpO_2_) [65]. The tHb response, a proxy measure of microvascular blood volume changes, was increased progressively in all studies [45,62,65]. Patients with mild cognitive impairment appear to have a smaller magnitude of increase in tHb than controls [65].

Summarizing the studies’ results, COPD patients exhibit lower cerebral oxygenation during exercise than healthy controls. This impairment is more severe in patients with excessive desaturation or those with comorbidities (HF) that exhibit blunted hemodynamic responses during exercise. As suggested by the above studies, an impairment in the autoregulation system (insufficient rise in MAP and/or CO) is one of the mechanisms contributing to the blunted cerebral oxygenation response during exercise. Indeed, it is well known that when mean arterial pressure (MAP) remains unchanged or decreases during exercise and/or there is a limited increase in cardiac output (CO), cerebral blood flow might be reduced, leading to impaired cerebral oxygenation [66,67,68]. Thus, it is conceivable that the coexistence of cardiac and pulmonary disease possibly leads to a low delivery of oxygen to the muscles and the CNS [68], causing more severe limitations during exercise in these patients [68]. An impaired cerebral oxygenation might exacerbate central fatigue, leading to early exercise intolerance [69]. Exercise capacity also seems to influence brain oxygenation response, as a link between cerebral oxygenation and peak exercise capacity in COPD with HF has been suggested [66].

### 4.2. Cerebral Oxygenation During Exercise in Patients with Interstitial Lung Disease (ILD)

Four studies explored the acute adaptations of cerebral oxygenation during exercise in patients with ILD and IPF, involving a total of 77 participants (Table 2). Two of these studies (Marillier et al. and Dipla et al.) examined patients with ILD/IPF both with and without significant exertional desaturation, using a cycling incremental exercise test [24,61]. The other two studies, conducted by the same research groups, investigated how supplemental oxygen influenced cerebral oxygenation during steady-state cycling at 60–65% of maximal workload [70,71]. Across all four studies, the results indicated that patients with ILD, particularly those with IPF, showed an inability to maintain cerebral oxygenation, even from the start of exercise.

In further detail, patients with significant exertional desaturation showed a decline in PFC oxygenation (as assessed by O_2_Hb) below resting levels from the beginning of the exercise session. This decline progressively worsened until the secession of exercise. Findings from both studies suggested that arterial desaturation during incremental cycling exercise is among the parameters, that may affect the magnitude of decline in cerebral desaturation. The study by Marillier et al. showed that patients with lower SpO_2_ had a larger fall in cerebral tissue saturation index (TSI) during exercise compared with control participants [24]. In addition, Dipla et al. reported that patients with exertional desaturation exhibited a significant decline in brain oxygenation from low exercise intensities (<40% of peak workload), whereas patients without significant desaturation experienced reduced cerebral oxygenation only at higher intensities (above 70% of peak work rate) [61]. It is worth mentioning that despite maintaining better arterial saturation, these patients still showed blunted cerebral O_2_Hb responses (compared to the responses expected in healthy controls). Based on the above, there might still be limitations in cerebral oxygenation during exercise even for IPF patients who maintain blood oxygen saturation levels, despite having a better response compared to the desaturation group [61].

Importantly, the studies by both Marillier et al. and Dipla et al. documented that the decline in cerebral oxygenation during exercise was linked to higher dyspnea (in Dipla et al., O_2_Hb response correlation with dyspnea rho = −0.71, *p* < 0.05 and shorter exercise duration (r = 0.42, *p* < 0.05), particularly in patients with exertional desaturation [24,61,70]. In fact, Marillier et al. also showed that greater cerebral deoxygenation (as assessed by the drop in TSI) was independently associated with lower exercise tolerance [24], whereas Dipla et al. observed an earlier exercise termination in patients with an O_2_Hb decline from the beginning of exercise [70]. Importantly, declines in cerebral TSI ≥ 4% were associated with lower predicted $\dot{V}$O_2_peak (r = 0.54, *p* < 0.05), and had poorer exercise tolerance and greater arterial desaturation [24,71]. The low fitness levels seem to also contribute to the cerebral responses in these patients, as the change in cerebral TSI during exercise was independently associated with $\dot{V}$O_2_peak [61].

In patients with significant desaturation, HHb values started to slightly increase from the first minute of exercise and increased even more at an exercise intensity of >25–40% [24,61,70,71]. In non-desaturators, HHb levels had a marked increase at higher relative intensities (>60–75% of peak exercise intensity) [61,71]. Lastly, tHb levels progressively increased during exercise showing no differences between desaturators and non-desaturators ILD patients or compared with healthy controls [24,61,70,71].

In summary, in patients with pulmonary fibrosis, and especially in patients with significant exertional desaturation, cerebral oxygenation was significantly impaired in a dose- and intensity-dependent fashion, directly affecting exercise tolerance and leading to early exercise termination. In the four studies, the tHb response was similar between IPF/ILD patients and healthy or IPF/ILD patients with and without arterial desaturation, suggesting that regional blood flow and blood vessel dilation in the brain’s microvasculature was not affected either by the presence of ILD or by the level of arterial desaturation [45]. Cerebral oxygenation plays a critical role in exercise performance for ILD patients. In conclusion, patients with ILD, and especially IPF, exhibit impaired cerebral oxygenation during exercise, reduced exercise tolerance, early exercise termination, and shortness of breath. Further research is needed to uncover the mechanisms driving these dysfunctions and evaluate whether exercise training interventions can mitigate them.

### 4.3. Cerebral Oxygenation During Exercise in Pulmonary Hypertension (PH)

Examining the literature for PH disease and brain oxygenation during exercise in adults, four studies (involving 51 patients in total) were identified (Table 3). Two studies compared cerebral oxygenation during exercise in PAH patients versus healthy controls [72,73], and two studies evaluated the effect of oxygen supplementation on cerebral oxygenation in this population [74,75]. In further detail, Boutou et al. (2021) implemented incremental cycling tests. The authors observed a decline in O_2_Hb below resting levels early in the exercise, with a limited increase in O_2_Hb at moderate intensities [74]. Reduced cerebral O_2_Hb was associated with hyperventilation and magnitude of oxygen desaturation at the end of the exercise session [73]. Across all four studies, HHb levels rose from the start of exercise; along with the low O_2_Hb, this finding indicates reduced brain oxygenation [72,73,74,75]. In addition, in Boutou et al.’s study (2021), autonomic nervous system dysfunction was noticed, including blunted baroreceptor sensitivity and parasympathetic activity, which likely contributed to poor exercise tolerance alongside cardiovascular issues [74]. Moreover, Malenfant et al. observed sustained reductions in cerebral TSI, accompanied by a parallel increase in cerebral HHB in PAH patients compared to controls [72]. Patients with significant exercise desaturation showed abnormalities in cerebrovascular responses, such as reduced middle cerebral artery flow, lower mean arterial pressure, decreased end-tidal CO_2_ (PETCO_2_, an index that reflects the ventilation–perfusion matching or mismatching within the pulmonary system [76]), and an elevated ventilatory equivalent for CO_2_ ($\dot{V}$E/$\dot{V}$CO_2_, a measure of ventilatory inefficiency and a predictor of respiratory complications [76,77]). The authors suggested that lower blood flow, hypoxemia and, eventually, impaired oxygenation to the brain during exercise collectively contribute to exercise intolerance in PAH patients [72]. Ulrich et al. showed that some of the deficits in cerebral oxygenation described above can be partially reversed through oxygen supplementation [75].

In conclusion, impaired cerebral oxygenation in PH patients during exercise appears to be associated with systemic and cerebrovascular dysfunctions, which, at least partially, contribute to exercise intolerance. Oxygen supplementation shows promise in improving brain oxygenation and could serve as a supportive therapy in managing PH-related exercise limitations.

### 4.4. Comparing Cerebral Oxygenation Deficits and Mechanisms in COPD, ILD, and PH During Exercise

**Figure 1:**
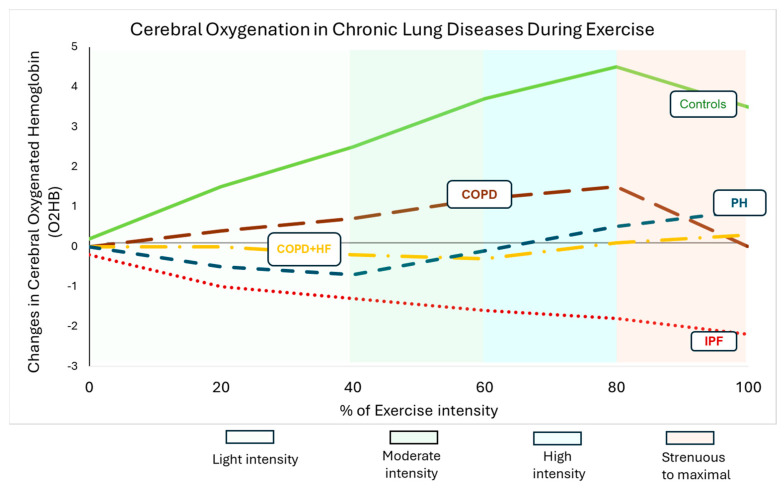
A schematic graph for cerebral oxygenation during exercise (maximal exercise testing on a bicycle ergometer) in chronic lung diseases and in healthy individuals. This representation was conducted after the literature review, and it is presented only for better understanding of the acute adaptations of cerebral oxygenated hemoglobin (O_2_Hb) during exercise. Individuals with chronic lung diseases, including COPD, ILD, and PAH, experience impaired cerebral oxygenation during exercise. The severity of this impairment is influenced by factors such as exercise intensity, comorbidities, hyperventilation, and exercise-induced desaturation. Patients with IPF with significant exercise desaturation exhibit the most pronounced cerebral oxygenation deficits. COPD patients with comorbidities or severe hypoxemia also show significant impairments, while PAH patients experience moderate reductions in brain oxygenation during exercise. Mechanisms behind these impairments include severe hypoxemia, disrupted cerebral autoregulation, hyperventilation, and altered hemodynamics, all of which contribute to decreased brain oxygen supply and exercise intolerance.

This review shows that individuals with different types of chronic lung diseases (such as COPD, ILD, PAH) experience impaired cerebral oxygenation during exercise. A schematic graph (Figure 1) illustrates these responses as reviewed in the literature. Patients IPF/fibrotic-ILD with significant exercise desaturation (SpO_2_ < 88% or a > 5% decline) exhibited the most pronounced reduction in brain oxygenation. Patients with PAH showed moderate decreases in cerebral oxygenation, less severe than in IPF, while in COPD patients cerebral oxygenation impairment was mainly observed in those with comorbidities or severe arterial hypoxemia. A consistent pattern in ILD and PAH patients was a gradual increase in HHb during exercise. This response was more pronounced in individuals with significant exercise desaturation. In COPD, cerebral oxygenation deficits were affected by exercise-induced desaturation and comorbidities.

**Figure 2:**
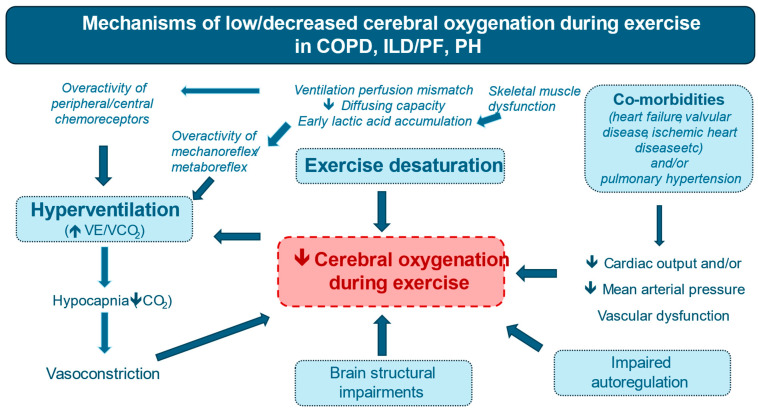
Potential mechanisms of blunted cerebral oxygenation during exercise in patients with Chronic obstructive pulmonary disease (COPD), Interstitial Lung Disease/Pulmonary Fibrosis (ILD/PF), and Pulmonary Hypertension (PH) compared with healthy individuals. Mechanisms behind these impairments include severe hypoxemia, hyperventilation, disrupted cerebral autoregulation, and altered hemodynamics, all of which contribute to decreased brain oxygen supply and contribute to exercise intolerance.

The underlying causes of impaired cerebral oxygenation in patients with chronic lung diseases remain a topic of research. Although the exact mechanisms are speculative, several factors have been described in previous studies that potentially contribute to reduced cerebral oxygenation during exercise in these patients (Figure 2) [24,61,72,73,78,79]. Excessive exercise-induced hyperventilation, as seen in more advanced stages of lung disease, can induce hypocapnia (a pronounced drop in CO_2_ levels). In this case, the brain may override its autoregulatory mechanisms and fail to increase blood flow adequately during exercise. Thus, hyperventilation-induced hypocapnia can cause cerebral vasoconstriction, leading to reductions in cerebral blood flow and oxygenation [45]. On the other hand, low brain O_2_ saturation might also stimulate excessive ventilation. Potential physiological mechanisms for this response include the following: (a) possible brain O_2_ sensors, (b) overstimulation of central and peripheral chemoreceptors, and (c) increased sensory feedback from group III/IV afferents from the active skeletal muscles (overstimulation of the exercise mechanoreflex/metaboreflex). In more detail, brain hypoxia sensors in the brainstem astrocytes can be activated under hypoxic conditions, resulting in rapid degradation of adenosine triphosphate in the brainstem. This, in turn, stimulates presympathetic neurons in the central respiratory circuit, leading to increased lung ventilation. Additionally, overactivity of both peripheral and central chemoreceptors in lung diseases might stimulate hyperventilation. Excessive signals from exercising muscles, due to the accumulation of metabolites, can increase sensory feedback from group III/IV skeletal muscle afferents and potentially further induce hyperventilation and increased sympathetic activity during exercise. Moreover, hypocapnia and early lactic acid accumulation during exercise might affect cerebral oxygenation by causing an early rightward and downward shift in the hemoglobin dissociation curve, which decreases O_2_ affinity for hemoglobin. This promotes greater O_2_ release in the cerebrovascular circulation, possibly reducing O_2_ content at the capillary level [80].

In support of these potential mechanisms, Malenfant et al. (2020) showed that patients with PAH had lower middle cerebral artery velocity (an index of cerebral blood flow) and lower cerebral oxygenation during an endurance exercise test compared to healthy controls. The significant correlations between cerebral oxygenation indices (HHb and Tissue oxygenation index), and end-exercise $\dot{V}$E/$\dot{V}$CO_2_ ratio (r = 0.50 and r = −0.52, respectively, *p* < 0.05), and SpO_2_ (r = −0.53, *p* < 0.05) suggest alterations in the cerebrovascular response to exercise in PAH [73]. Furthermore, in patients with IPF, Dipla et al. [61] also reported that the average cerebral O_2_HB response during exercise was correlated with dyspnea, (rho = −0.71, *p* < 0.05), cardiopulmonary exercise testing duration (r = 0.42, *p* < 0.05), % predicted DLCO (r = 0.53, *p* < 0.05), and 6 min walking test distance (r = 0.58, *p* < 0.05).

Co-morbidities, such as heart failure or valvular disease, cause hemodynamic alterations (low mean arterial pressure and/or cardiac output) that can also exacerbate the reductions in cerebral blood flow, compounding oxygenation issues [66,67,68]. These mechanisms collectively may explain the observed declines in cerebral oxygenation and their impact on neuronal function and exercise performance in patients with chronic lung diseases [51]. Inadequate brain oxygenation during exercise can lead to progressive cortical dysfunction central command, resulting in increased central fatigue and reduced muscle force, and ultimately exercise intolerance [42,81].

### 4.5. Methodological Limitations and Bias of Reported Results

The studies presented in this review have some methodological limitations and potential biases that may influence the reported results: (i) Many studies had relatively small sample sizes and wide age ranges. Additionally, the presence of co-morbidities in patients may confound the results, as these factors can independently impact cerebral oxygenation and exercise performance. (ii) Some studies lacked a control group, making it difficult to directly compare the results to healthy individuals. (iii) There was inconsistency in the variables reported across studies. Some studies based their conclusions on measurements of oxygenated hemoglobin (O_2_Hb), deoxygenated hemoglobin (HHb), or total hemoglobin (tHb), while others relied on the Tissue Oxygenation Index (TSI or TOI) or HHb response alone. (iv) One positive aspect is that all studies used a common exercise modality—bicycle ergometry—because exercise modality (bicycle vs. treadmill) can influence cerebrovascular responses to exercise [82]. However, there was variability in the exercise protocols used. Some studies focused on maximal exercise testing, while others used submaximal steady-state tests, and a few employed both maximal and submaximal steady-state protocols. In addition, the majority of studies used the Oxymon Mk III device (Artinis), with a few employing the NIRO-300 (Hamamatsu Photonics). The sampling rates varied from 1 to 10 Hz, which may lead to differences in the precision and reliability of the measurements across studies. (v) Most studies assessed only the prefrontal cortex, with a small number examining multiple brain regions. Therefore, no conclusions can be drawn about oxygenation in other brain areas, limiting the understanding of cerebral oxygenation across the entire brain during exercise. (vi) All studies found in the literature assessed only acute adaptations to exercise, without examining responses following treatment (whether pharmacological or non-pharmacological, such as exercise training). This limits the ability to understand long-term effects or adaptations to exercise and treatment over time. Additionally, while a few studies investigating oxygen supplementation during exercise have shown improvements in brain oxygenation and prolonged exercise time, the long-term effects of oxygen supplementation on brain oxygenation and exercise capacity have not been explored. Despite these methodological differences and limitations, all studies consistently agree that cerebral oxygenation during exercise is compromised in patients with chronic lung diseases (such as COPD, ILD, and PH) compared to healthy controls. Further research is necessary to gain a deeper understanding of the cerebral oxygenation impairments during exercise in chronic lung disease and to explore potential interventions that could improve these dysfunctions.

## 5. Conclusions

This review highlights that individuals with chronic lung diseases, including COPD, ILD, and PAH, experience impaired cerebral oxygenation during exercise. The severity of this impairment is influenced by factors such as exercise intensity, comorbidities, and exercise-induced desaturation. Patients with IPF with significant desaturation exhibit the most pronounced cerebral oxygenation deficits. COPD patients with comorbidities or severe hypoxemia show similar impairments, while PAH patients experience moderate reductions. Mechanisms behind these impairments include severe hypoxemia, disrupted cerebral autoregulation, hyperventilation, and altered hemodynamics, all of which contribute to decreased brain oxygen supply and exercise intolerance. Further studies should explore whether improving cerebral oxygenation through exercise training or in combination with medications (e.g., antifibrotic medication, etc) is feasible. Such research could provide insights into potential therapeutic strategies to enhance brain oxygen supply and improve exercise capacity and overall quality of life in patients with chronic lung diseases.

## Figures and Tables

**Table 1:** Studies in patients with Chronic Obstructive Pulmonary Disease (COPD) examining cerebral oxygenation during exercise using NIRS.

Author(s)	Participants/ Age (Yrs)	Type of Exercise	Results	Conclusions
Higashimoto et al., 2011 [62]	10 COPD (70.6 ± 2.7 yrs) 10 controls (69.0 ± 1.8 yrs) SpO_2_ nadir NR	Steady state test on a cycle ergometer (at 40% of WRpeak for ~10 min)	PFC: O_2_Hb gradually increased, with a significant increase at the 9th or 10th min of exercise. HHb tended to decrease after the first minutes of exercise and tHb tended to increase. TPC: there were no significant changes in all NIRS parameters during exercise. A tendency for smaller increase in O_2_Hb and tHb in COPD patients than controls; however, these differences were NS. Hbdiff: NR	Significant activation of PFC during exercise, whereas the temporoparietal regions were not significantly activated Exertional dyspnea was related to PFC oxygenation (changes in O_2_Hb) in COPD patients and in control participants
Oliveira et al., 2012 [63]	8 COPD desaturators (66.7 ± 7.9 yrs) SpO_2_ nadir 86 ± 3% 12 non desaturators (60.3 ± 5.9 yrs) SpO_2_ nadir 96 ± 2%	Incremental cycling to exhaustion (10 Watts/min)	O_2_Hb: in desaturators, not significantly altered till late exercise; in non desaturators, it increased progressively from moderate exercise (~50–60% WRpeak); it was significantly lower in desaturators than non-desaurators. HHb: NR, tHb: NR, Hbdiff: NR	COPD patients with exertional desaturation had impaired cerebral oxygenation in a normoxic environment.
Oliveira et al., 2013 [66]	15 COPD (65 ± 8 yrs) SpO_2_ nadir 90 ± 6% 18 COPD + HF (67 ± 7 yrs) SpO_2_ nadir 93 ± 3%	Incremental cycling to exhaustion (Watts NR)	O_2_Hb: in COPD, it increased in 11/15 patients; in COPD + HF, it remained stable or decreased in 14/18 patients; in cases where mean systemic arterial pressure remained stable or decreased, O_2_Hb was impaired. HHb: NR, tHb: NR, Hbdiff: NR	In patients with moderate-to-severe COPD, comorbidities, such as HF, worsen cerebral oxygenation during exercise
Vogiatzis et al., 2013 [64]	12 COPD (66 ± 5 yrs) SpO_2_ nadir 88 ± 4%	Steady state exercise to exhaustion (75% WRpeak) breathing (i) room air, (ii) pure oxygen, and (iii) normoxic heliox	CBF increased from rest to exhaustion. At exhaustion, CBF was higher while breathing air and heliox than oxygen. Cerebral oxygenation (StO_2_): at rest, it was lower in air or heliox than in oxygen. During exercise: when breathing air, StO_2_ did not change from rest and was lower at exhaustion and isotime than with supplemental oxygen. Time to exhaustion while breathing air was less than with oxygen or heliox supplementation. HHb: NR, tHb: NR, Hbdiff: NR	Breathing oxygen and heliox prolonged time to exhaustion, StO_2_ was lower with heliox than oxygen, but patients had similar endurance time. StO_2_ was similar in air and heliox despite greater endurance with heliox.
Oliveira et al., 2016 [67]	10 COPD SpO_2_ nadir 87 ± 5% (65 ± 8 yrs) 8 COPD + HF (65 ± 6 yrs) SpO_2_ nadir 93 ± 3%	Cycling (at 20%, 40%, 60% and 80% WRpeak, 4 min stages at each intensity)	O_2_Hb: in COPD, it increased by ~ 10 μmol/s from rest to 80% WRpeak (*p* < 0.05). In COPD + HF, it was below baseline from the first minutes of exercise. CBF: was significantly lower in COPD + HF than in COPD patients. CBF increased by ~40% during exercise, whereas it was reduced ~10% in COPD + HF HHb: NR, tHb: NR, Hbdiff: NR	Cerebral oxygenation and CBF during exercise were lower in COPD with HF than COPD. Combined effects of systemic hemodynamic impairements (reduced CO and MAP) and lower PaCO_2_ might be responsible for the limited increase in cerebral oxygenation during exercise.
Higashimoto et al., 2015 [45]	11 COPD hypoxemic (71.2 ± 2.1 yrs)SpO_2_ nadir 86 ± 1.1% 16 COPD non hypoxemic (72.9 ± 1.7 yrs) 11 controls (70.5 ± 2.4 yrs)	Steady state cycling (at 40% of WRpeak)	O_2_Hb: in hypoxemic COPD, it decreased at the beginning of exercise (1–5 min) and then slowly increased (8–10 min). O_2_Hb was significantly lower in hypoxemic than non-hypoxemic and controls (at 2–9 min). In non hypoxemic COPD and control groups, it increased during exercise. HHb: in hypoxemic, it increased during exercise; in non- hypoxemic and control groups, it decreased during exercise; it was significantly higher (at 3–8 min) in hypoxemic than non-hypoxemic and control. tHb: it increased during exercise, but no significant differences occurred between groups.	Impaired cortical oxygenation during exercise in COPD patients with hypoxemia vs. control and non-hypoxemic patients. Exertional dyspnea was associated with activation of the pre-motor cortex in non-hypoxemic and control participants, whereas it was associated with increased HHb of the PFC in hypoxemic patients.
Andrianopoulos et al., 2018 [65]	31 COPD+ CN (68.5 ± 9.1 yrs) SpO_2_ nadir 92.3 ±2.7 21 COPD+ CI (67.8 ± 9.0 yrs) SpO_2_ nadir 90.3 ±3.0	Steady state cycling (at 75% of WRpeak)	tHb: there was an increase from baseline to the limit of tolerance in both CN and CI patients. There was a comparable change in O_2_Hb and HHb in CN and CI patients from baseline to the limit of tolerance. Cerebral TSI remained unchanged during exercise in both groups.	COPD patients with CI had a similar cerebral oxygenation response during submaximal exercise to patients with CN.
da Luz Goulart et al. 2021 [68]	11 COPD + HF (69 ± 7 yrs) SpO_2_ nadir 93.1 ± 0.1 11 HF (62 ± 6 yrs)	Steady state cycling to exhaustion (at 80% of WRpeak)	O_2_Hb: in COPD + HF, it declined faster than in the HF group. HHb: there was a lower increase in COPD + HF than in HF, although similar slope of increase. tHb:NR Hbdiff:NR	The coexistence of COPD and HF can negatively affect cerebral oxygenation and result in greater exertional dyspnea, possibly contributing to lower exercise tolerance.

Abbreviations: PFC—prefrontal cortex; TPC—temporoparietal cortex; HF—heart failure; CBF—cerebral blood flow; MAP—mean arterial pressure; CI—cognitive impairment; CN—cognitive normal; O_2_Hb—oxygenated hemoglobin; HHb—deoxygenated hemoglobin; Hbdiff—hemoglobin difference; tHb—total hemoglobin; SpO_2_—desaturation; TSI—tissue saturation index; WRpeak—work rate peak; NR—not reported; NS—non-significant.

**Table 2:** Studies in patients with fibrotic Interstitial Lung Disease (ILD) and cerebral oxygenation during exercise using NIRS.

Author(s)	Participants Age (Yrs)	Type of Exercise	Results	Conclusions
Dipla et al., 2023 [61]	23 ILD (IPF) 13 IPF with exertional desaturation (62.9 ± 10.0 yrs) SpO_2_ nadir 82.6 ± 3.9% 10 non-exertional desaturation (60.8 ± 8.6 yrs) SpO_2_ nadir 92.3 ± 2.5%	Incremental cycling: workload increments 10 W/min, at 50 rpm	O_2_Hb: in patients with exertional-desaturation*,* there was no increase in O_2_Hb from the beginning of exercise, and it significantly declined during the session; in patients without exertional-desaturation, O_2_Hb remained above baseline levels throughout the exercise session. There were lower responses in desaturators compared to non-desaturators. O_2_Hb responses significantly correlated with DLCO (r = 0.53, *p* < 0.01) HHb: in patients with exertional desaturation*,* HHb progressively increased at intensities >50% of $\dot{V}$O_2_peak; in patients without-desaturation*,* it significantly increased only at intensities >80% of $\dot{V}$O_2_peak. Higher responses were observed in desuturators compared to non-desuturators. Hbdiff: in patients with exertional-desaturation*,* Hbdiff significantly decreased at 50% $\dot{V}$O_2_peak and continued to decline until maximum exercise; in non-desaturators*,* Hbdiff remained close to baseline levels and decreased only at max exercise. There were lower responses in desuturators than non-desaturators. tHb: there was a progressive increase during exercise, but no differences were evident in tHb responses between groups.	Patients with IPF and isolated exertional desaturation presented significantly lower cerebral oxygenation compared to those without exertional desaturation. The decline in brain oxygenation in the former patients begins at low exercise intensities (<40% of $\dot{V}$ O_2_peak) and can be markedly reduced at intensities >50% of $\dot{V}$O_2_peak. Lower brain oxygenation was significantly associated with shorter exercise duration, greater dyspnea, and more severe diffusion limitations (lower DLCO).
Marillier et al., 2021 [24]	27 ILD (72 ± 8 yrs) 16 patients with cerebral deoxygenation (SpO_2_ nadir 81.7 ± 8.0%) 11 patients without cerebral deoxygenation (SpO_2_ nadir 90.4 ± 3.0%) 12 Healthy controls (73 ± 9 yrs)	Incremental cycling symptom limited test (workload increments 5–15 watt every 2 min)	O_2_Hb: in patients with cerebral deoxygenation, O_2_Hb declined from the beginning of exercise, showing a significant decline compared to baseline at intensities >60% of exercise duration. HHb: in patients with cerebral deoxygenation, HHb progressively increased from the start of exercise; significantly higher than baseline at intensities >40% of exercise duration. In patients without cerebral deoxygenation and controls*,* HHb remained relatively unchanged. tHb: no significant differences among groups. Hbdiff: NR.	ILD patients with exertional desaturation exhibited a dose-dependent relationship with reduced cerebral oxygenation. Exercise desaturation may impair cerebral oxygenation, leading to exercise intolerance in those patients.
Dipla et al., 2021 [70]	13 ILD (IPF) (63 ± 10 yrs) SpO_2_ nadir 83.5 ± 3.6%	Steady state cycling: exercise (at 65% of WRpeak) Exercised breathing (i) medical air and (ii) supplemental oxygen	Under room air O_2_Hb: it significantly declined from the beginning of exercise and remained low. HHb: It progressively increased from the start of exercise. Hbdiff: it remained below pre-exercise values throughout exercise. tHb: it showed a progressive increase from the start of exercise. Patients with greater decline in O_2_Hb and Hbdiff from the initial minutes of exercise were the ones to terminate exercise earlier (<5 min).	IPF patients with isolated exertional desaturation exhibited an inability to maintain or increase cerebral oxygenation during submaximal exercise. Patients who exhibited a decline in O_2_Hb from the beginning of exercise terminated exercise earlier and presented significantly more dyspnea. O_2_ supplementation increased brain oxygenation and prolonged exercise duration.
Marillier et al., 2023 [71]	14 fibrotic (f- ILD; 8/14 IPF (72 ± 8 yrs) ΔSpO_2_—13% 14 healthy controls (73 ± 8 yrs) ΔSpO_2_—1%	Steady state cycling (60% of WRpeak) to symptom limitation Exercised breathing (i) medical air and (ii) supplemental oxygen	O_2_Hb:in ILD patients, it declined from pre-exercise values during the exercise test; ILD patients had significantly lower responses than controls in the air protocol. HHb: in ILD patients there was a significant increase in the air protocol. Hbdiff: in ILD patients there was a significant decline from the baseline value and it remained constantly below baseline in the air protocol. tHb: there was no significant difference between groups in the overall response.	Patients with severe exertional hypoxemia (80 ± 8%) had poorer cerebral oxygenation and greater fatigue compared to controls when breathing room air. Supplemental oxygen improved arterial hypoxemia, bringing brain oxygenation closer to the response observed in control individuals. There was a significant correlation between HbDiff responses and the rate of perceived fatigue throughout exercise in f-ILD (repeated-measures correlation = −0.51, *p* < 0.001).

Abbreviations: IPF—idiopathic pulmonary fibrosis; NIRS—near-infrared spectroscopy; SpO_2_—O_2_ saturation by pulse oxymetry; WRpeak—work rate peak; O_2_Hb—oxygenated hemoglobin; HHb—deoxygenated hemoglobin; Hbdiff—hemoglobin difference; tHb—total hemoglobin; ΔSpO_2_—change in oxygen pulse saturation during exercise; $\dot{V}$O_2_peak—peak oxygen uptake; WRpeak—work rate peak; NR—not reported.

**Table 3:** Studies in patients with Pulmonary hypertension (PH) examining cerebral oxygenation during exercise using NIRS.

Author(s)	Participants Age (Yrs)	Type of Exercise	Results	Conclusions
Malenfant et al., 2017 [72]	11 PAH (44 ± 12 yrs) 11 healthy controls (43 ± 15 yrs)	Incremental cycling (5–25 W/min until exhaustion)	In PAH patients low cerebral oxygenation, during early exercise and it remained low throughout the exercise session. HHb: it gradually increased from the beginning of exercise. tHb: NR, Hbdiff: NR	In PAH patients, during exercise there was lower cerebral oxygenation compared to controls and a sustained decrease in cerebral TOI during exercise. Lower TOI and increased HHb correlated with maximal exercise capacity (r = 0.52 and 0.54, respectively, *p* < 0.01)) in patients with PAH. there was impaired cerebral hemodynamic regulation and oxygenation
Ulrich et al. 2017 [75]	22 PAH/CTEPH (61 ± 14 yrs) SpO_2_ nadir 89.9 ± 7.9%	Incremental cycling to exhaustion (10–20 W/min 50–60 rpm steady state test (at 75% WRpeak)	Cerebral tissue oxygenation did not present an increase during exercise. O_2_Hb, tHb, Hbdiff: NR	In PAH/CTEPH patients, brain oxygenation did not present the expected increase during exercise when breathing oxygen-enriched air. Oxygen supplementation improved brain oxygenation, enhanced ventilatory efficiency, and increased exercise performance in PAH patients.
Malenfant et al., 2020 [73]	9 PAH (45 ± 12 yrs) 10 healthy controls (44 ± 15 yrs)	Steady state cycling (at 75% of the WRpeak, 60 rpm)	Cerebral tissue oxygenation index (ΔcTOI): in PAH patients, it decreased throughout the endurance cycling test, whereas it remained unchanged in controls. HHb: it gradually increased during exercise, marginally more compared to controls. O_2_Hb: NR, tHb: NR, Hbdiff: NR	A significant correlation was found between markers of cerebral oxygenation (HHb and TOI), and end-exercise $\dot{V}$E/$\dot{V}$CO_2_ ratio (r =0.50 and r = −0.52, respectively, *p* < 0.05), suggesting that low brain O_2_ saturation might stimulate excessive ventilation in PAH. Lower MCAv and cerebral oxygenation during endurance exercise were observed in PAH compared to controls.
Boutou et al., 2021 [74]	9 PAH (51.4 ± 9.4 yrs) SpO_2_ nadir 87.6 ± 3.7 With medical air or oxygen supplementation	Steady state cycling (at 65% of WRpeak, 50–60 rpm)- effects of acute oxygen supplementation	O_2_Hb: below baseline at the start of exercise, there was a small increase over time. HHb: it increased from the start of exercise. Hbdiff: it remained below baseline from the start of exercise and throughout the session. tHb: it increased during exercise. TSI: it decreased (by 14%) in the air protocol.	PAH patients presented low PFC oxygenation during exercise when breathing room air, as well as low cardiac output and autonomic dysfunction (blunted responses in Baroreceptor sensitivity). In PAH/CTEPH patients, oxygen supplementation during submaximal significantly improved cerebral oxygenation, prolonged exercise time, increased cardiac output, and improved autonomic function without significantly affecting muscle oxygenation.

Abbreviations: PFC—prefrontal cortex; MCAv—middle cerebral artery; NIRS—near-infrared spectroscopy; SpO_2_ nadir—lowest dioxygen pulse saturation during exercise; O_2_Hb—oxygenated hemoglobin; HHb—deoxygenated hemoglobin; Hbdiff—hemoglobin difference; tHb—total hemoglobin; $\dot{V}$O_2_peak—peak oxygen uptake; WRpeak—work rate peak; NR—not reported, TOI—tissue oxygenation index.

## Glossary

CNS	Central nervous system
COPD	Chronic Obstructive Pulmonary Disease,
CTEPH	Chronic Thromboembolic Pulmonary Hypertension,
DLCO	Diffusing lung capacity for carbon monoxide
HHb	Deoxygenated hemoglobin
Hbdiff	Hemoglobin difference
ILD	Interstitial Lung Disease
IPF	Idiopathic pulmonary fibrosis
NIRS	Near-Infrared-Spectroscopy
O_2_Hb	Oxygenated hemoglobin
PAH	Pulmonary arterial hypertension
PETCO_2_	End-tidal CO_2_
PH	Pulmonary Hypertension
PFC	Prefrontal cortex
SpO_2_, %	Oxygen hemoglobin saturation from pulse oxymetry
tHb	Total hemoglobin
TSI, %	Tissue saturation index
$\dot{V}$E/$\dot{V}$CO_2_ ratio	Ventilatory equivalent for CO_2_ (i.e., Minute ventilation-to-carbon dioxide output)
